# DCI after Aneurysmal Subarachnoid Hemorrhage Is Related to the Expression of MFG-E8

**DOI:** 10.1155/2021/6568477

**Published:** 2021-12-31

**Authors:** Xianjun Chen, Yong'an Jiang, Jiayu Liu, Changfeng Wang, Dengfeng Wan, Ai'jun Liang, Jingxing Leng, Yu Yang, Hui Xiang, Ru'en Liu

**Affiliations:** ^1^Department of Neurosurgery, Jiangxi Provincial People's Hospital Affiliated to Nanchang University, Nanchang, Jiangxi 330006, China; ^2^Department of Neurosurgery, Peking University People's Hospital, 11th Xizhimen South St. Beijing 100044, China

## Abstract

**Objective:**

To explore the predictive value of milk fat globule epidermal growth factor 8 (MFG-E8) in the occurrence of delayed cerebral ischemia (DCI) after an aneurysmal subarachnoid hemorrhage (aSAH).

**Methods:**

We recruited 32 patients with aSAH as the case group and 24 patients with unruptured aneurysms as the control group. Serum MFG-E8 levels were measured by western blot and enzyme-linked immunosorbent assay. We analyzed the relationship between MFG-E8 levels and the risk of DCI.

**Results:**

The levels of serum MFG-E8 in the case group (mean = 11160.9 pg/mL) were significantly higher than those in the control group (mean = 3081.0 pg/mL, *p* < 0.001). MFG-E8 levels highly correlated with the World Federation of Neurosurgical Societies (WFNS) and modified Fisher scores (*r* = −0.691 and − 0.767, respectively, *p* < 0.001). In addition, MFG-E8 levels in patients with DCI (5882.7 ± 3162.4 pg/mL) were notably higher than those in patients without DCI (15818.2 ± 3771.6 pg/mL, *p* < 0.001). A receiver operating characteristic curve showed that the occurrence of DCI could effectively be predicted by MFG-E8 (area under the curve = 0.976, 95%CI = 0.850–1.000). Kaplan–Meier survival analysis showed a remarkable decrease in the incidence of DCI in case group individuals with high levels of MFG-E8 (≥11160.9 pg/mL, *p* < 0.001).

**Conclusion:**

MFG-E8 may be a useful predictive marker for DCI after an aSAH and could be a promising surrogate end point.

## 1. Introduction

Aneurysmal subarachnoid hemorrhage (aSAH) is an acute and critical disease with high morbidity and mortality [1–4]. Some surviving patients show clear cognitive impairment or even lifelong disability [5].

After aSAH, a cerebral vasospasm can effectively decrease blood flow and result in delayed cerebral ischemia (DCI). DCI, also known as symptomatic cerebral vasospasm, is a common complication usually occurring 3–14 days after aSAH and is considered to be the main cause of poor prognoses [6–8]. Therefore, in the diagnosis and treatment of aneurysms, early detection and prevention of DCI could help improve the patient's quality of life and relieve the clinical burden [9]. At present, the pathogenesis of DCI is unclear. Previous studies have found some evidence for the participation of cerebral vasospasm, microthrombosis, and inflammation in the pathophysiological processes of DCI [10–14].

Milk fat globule epidermal growth factor 8 (MFG-E8), also known as milk lectin, is expressed in many organs throughout the body. It is a bridge between apoptotic cells and phagocytes [15, 16]. It plays an important role in maintaining vascular integrity, essentially inhibiting inflammation and microthrombosis [17–20]. We hypothesize that MFG-E8 levels may be related to the pathogenesis of DCI. This is the first study to describe the relationship between serum MFG-E8 levels and DCI in patients with aSAH. The present study also discusses the potential of MFG-E8 markers in predicting DCI after aSAH.

## 2. Materials and Methods

### 2.1. Chemicals, Reagents, and Antibodies

Antibodies against *β*-actin and *β*-tubulin were purchased from the Proteintech Group (Wuhan, China). MFG-E8 antibody was purchased from Biorbyt (Cambridge, United Kingdom). An enzyme-linked immunosorbent assay (ELISA) kit was purchased from Servicebio (Wuhan, China).

### 2.2. Sample Collection

As a prospective cohort study without planned intervention, we divided the subjects into a case (aSAH group) and a control group (non-aSAH group). The case group included 32 aSAH patients of the department of neurosurgery at the Jiangxi Provincial People's Hospital from September 2020 to December 2020. In addition, 24 patients with unruptured aneurysms, hospitalized during the same period, were included as the control group. The clinical baseline information of patients was retrospectively collated from the hospital's electronic medical system and verified by trained medical staff.

Fasting blood was collected by a separate gel coagulation-promoting vacuum tube on the morning of the second day after admission. After allowing the sample to coagulate for 15~30 min, the blood samples were centrifuged at 2500 r/min for 10 min, and the serum was collected and stored at -80°C. The study design conformed to the guidelines outlined in the Helsinki Declaration and was approved by the Research Ethics Committee of Jiangxi Provincial People's Hospital (Approval number: 2021-78). All participants or representatives provided informed consent.

### 2.3. ELISA

Serum MFG-E8 levels were determined using an ELISA (EK-H11462, Servicebio) according to the manufacturer's instructions. Sample concentrations were calculated from a standard curve, fitted using a quadratic polynomial regression equation.

### 2.4. Western Blotting

Western blotting was performed using the serum of both study groups. The serum was diluted to a 20% concentration of the original, and a 5x loading buffer (Servicebio) was added. Equal amounts of total protein were separated using 10% sodium dodecyl sulfate-polyacrylamide gel electrophoresis and transferred to polyvinylidene difluoride membranes (Millipore, China). The membranes were blocked with 5% bovine serum albumin at room temperature for 1 h and incubated with specific primary antibodies overnight at 4°C. The appropriate secondary antibodies conjugated with horseradish peroxidase were incubated for 1 h at room temperature. The western blot signal was obtained using Super Signal ECL (Zen Bio, Chengdu, China).

### 2.5. Clinical Evaluation and Follow-Up

The subarachnoid blood volume was measured using computed tomography (CT) scan and semiquantified using modified Fisher scores [21, 22]. The severity of aSAH was assessed according to the rating of the World Federation of Neurosurgical Societies (WFNS) [23]. Each patient's condition was graded by the same neurosurgeon.

Patients in the case group were treated by the same neurosurgeon, including surgical intervention and prescription of medication during the treatment phase. Brain edema and intraparenchymal hematoma were determined using the CT scan, and the location and size of each aneurysm were determined by computed tomography angiography (CTA) or digital subtraction angiography (DSA).

Follow-ups for patients in the case group were conducted at 3 months to determine the occurrence of DCI events after aSAH.

### 2.6. Inclusion and Exclusion Criteria

The inclusion criteria of the case group were as follows: (1) admission within 24 h after onset of aSAH, (2) patients with aSAH caused by intracranial aneurysms confirmed by CTA or DSA, (3) clipping of intracranial aneurysms within 48 h after admission, and (4) aSAH treated according to the guidelines of the American Heart Association and American Stroke Association [24, 25].

The exclusion criteria were as follows: (1) admission more than 48 h after onset; (2) death occurred immediately after admission; (3) patients with chronic malnutrition, malignant tumors, and autoimmune diseases; (4) all patients with severe cardiovascular diseases such as coronary atherosclerosis; and (5) patients with rebleeding after more than one ruptured aneurysm.

### 2.7. Diagnosis of DCI

The diagnosis of DCI was based on evaluation by a clinician and imaging results. The evaluation excludes other causes where, (A) for example, hydrocephalus, rebleeding, or epilepsy caused a new focal functional neurological impairment (hemiplegia, aphasia, hemianopsia, or neglect) that lasts for at least 1 h; (B) the Glasgow coma scale score is reduced by 2 points or more for at least 1 h and cannot be attributed to other reasons; (C) radiological signs of infarction are confirmed by one of the following criteria: (1) seen >48 h after any intracranial procedure and absent on intervening imaging; (2) seen >48 h after any intracranial procedure without intervening imaging in a location distant from the operative site and not considered to be caused by a procedure (e.g., an embolic complication of angiography); (3) seen <48 h after an intracranial procedure in the setting of a severe vasospasm; and (4) seen <48 h after an intracranial procedure in a vascular or watershed territory distinct from the operative site, not considered to be caused by a procedure (e.g., angiography) [26–30].

### 2.8. Statistical Analyses

R v4.0.4, SPSS v17.0, Medcalc v19.8, and GraphPad Prism v8.0 software were used for all statistical analyses. A Shapiro–Wilk test was used to test the normality of continuous variables. The results for normally distributed data are presented as mean (standard deviation or SD), and those of nonparametric data are reported as median (interquartile range or IQR). The categorical variables are expressed by count (percentage) and analyzed using the Pearson *χ*^2^ test or Fisher exact test. According to WFNS scores and modified Fisher scores, levels of MFG-E8 were divided into five grades: grade 1 (>20,000 pg/mL), grade 2 (15,000~20,000 pg/mL), grade 3 (10,000~15,000 pg/mL), grade 4 (5,000~10,000 pg/mL), and grade 5 (<5,000 pg/mL). A logistic regression model was used to evaluate the potential predictors of DCI events after aSAH. A *Z* test was used to evaluate the performance of an area under the curve (AUC) model. The sensitivity, specificity, and Jordan index of WFNS scores, modified Fisher scores, and MFG-E8 levels were described using a receiver operating characteristic curve of subjects' working characteristics, and the best threshold for judging the occurrence of DCI was subsequently obtained.

We divided the MFG-E8 levels in the aSAH group into high-level (≥12500 pg/mL) and low-level (<12500 pg/mL) groups according to the mean to analyze the relationship between MFG-E8 level and DCI events after aSAH using Kaplan–Meier survival curves. In all analyses, statistical significance was set to *p* < 0.05.

## 3. Results

### 3.1. Patient Characteristics

The 32 patients of the case group included 11 males and 21 females, with a median age of 56.5 (48.0–63.0) years. The 24 patients comprising the control group included 16 males and 8 females, with a median age of 60.5 (55.5–68.0) years (Table 1).

**Figure 1:**
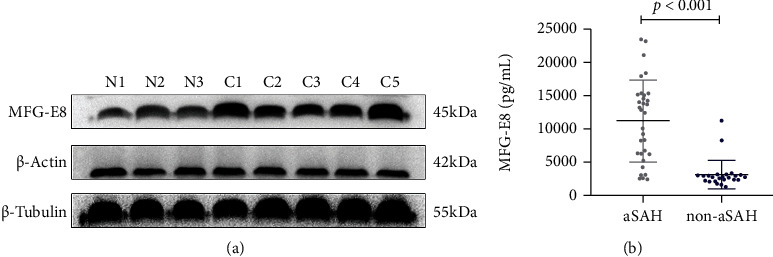
MFG-E8 levels in the aSAH and the non-aSAH groups. (a) Three patients were randomly selected from the non-aSAH group (N1~N3), and five patients were randomly selected from the aSAH group (C1~C5). The expression of MFG-E8 in the aSAH group was significantly higher than that in the control group. (b) ELISA was performed on the serum samples of all patients in both groups, and the results were consistent with those obtained using western blotting; the expression of MFG-E8 increased significantly in the case group.

Between these groups, there were significant overall differences in MFG-E8 levels (11160.9 ± 6102.0 pg/mL vs. 3081.0 ± 2151.1 pg/mL, *p* < 0.001) and overall aneurysm diameter (*p* < 0.05). There was also a significant difference in MFG-E8 levels between sexes within the groups (*p* < 0.05) (Figure 1).

### 3.2. MFG-E8 Levels in the aSAH and DCI Groups

**Figure 2:**
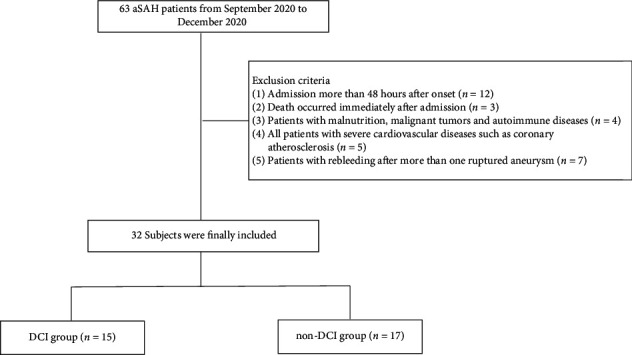
Flowchart of enrolled subjects.

The demographic, clinical, laboratory, and radiological data were analyzed for all the patients in our dataset. Accounting for both groups, 21 (65.6%) were female of median age 56.5 (48.0–63.0) years. Median aneurysm size was 5.0 (4.0–6.0). Cystic aneurysms accounted for 50% (16/32) of all cases. Of all the patients, acute hydrocephalus was observed in 17 (53.1%) and DCI in 15 (46.9%). The average MFG-E8 level was 11160.9 pg/mL (6102.0) (Table 2). During the 3-month follow-up, patients in the aSAH group were divided into a DCI group and a non-DCI group (Figure 2).

**Figure 3:**
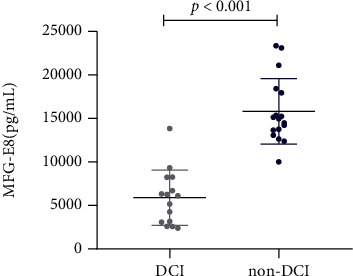
MFG-E8 levels in the DCI and non-DCI groups.

We compared the demographic, clinical characteristics, and MFG-E8 levels of patients with DCI (*n* = 15) and those without DCI (*n* = 17) after aSAH (Table 3). The MFG-E8 level of the DCI group (5882.7 ± 3162.4 pg/mL) was significantly lower than that of the non-DCI group (15818.2 ± 3771.6 pg/mL, *p* < 0.001) (Figure 3).

WFNS and modified Fisher scores are highly correlated with the severity of aSAH and are clinically used to predict the occurrence of DCI. The WFNS and modified Fisher scores of the DCI group were remarkably higher than those of the non-DCI group (*p* < 0.001) (Table 3).

**Figure 4:**
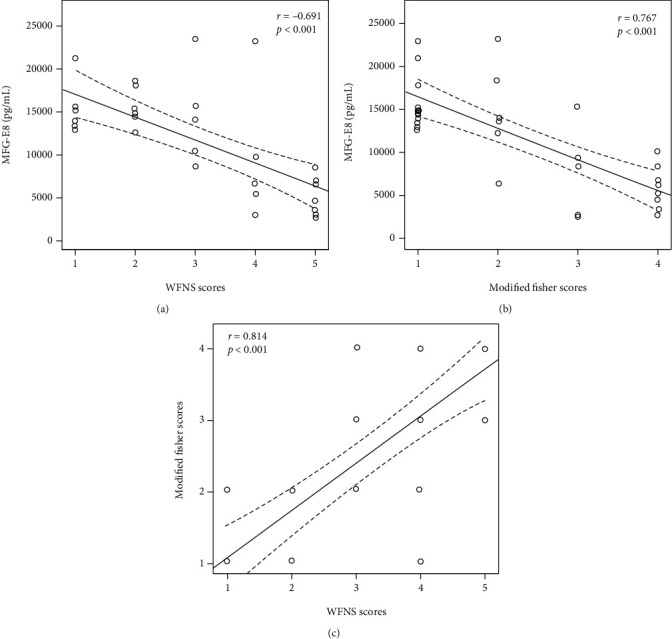
Relationship between biomarkers and clinical scores in patients with aSAH. Relationship between (a) WFNS scores and MFG-E8, (b) modified Fisher scores and MFG-E8, and (c) WFNS and modified Fisher scores.

According to the Pearson correlation analysis, the serum MFG-E8 level strongly correlated with the WFNS and modified Fisher scores at admission (*r* = −0.691 and − 0.767, respectively, *p* < 0.001) (Figure 4).

We found that, except for MFG-E8 level, hypertension, WFNS scores, and modified Fisher scores were all closely related to the occurrence of DCI (Table 4).

The AUC of WFNS scores and MFG-E8 were 0.973 (95%CI = 0.844, 1.000, *Z* = 0.149) and 0.976 (95%CI = 0.850, 1.000, *Z* = 0.882), respectively. The AUC of the improved modified Fisher scores was 0.927 (95%CI = 0.778 ~ 0.989, *Z* = 1.093) compared with the AUC of MFG-E8 (*Z* = 0.274).

**Figure 5:**
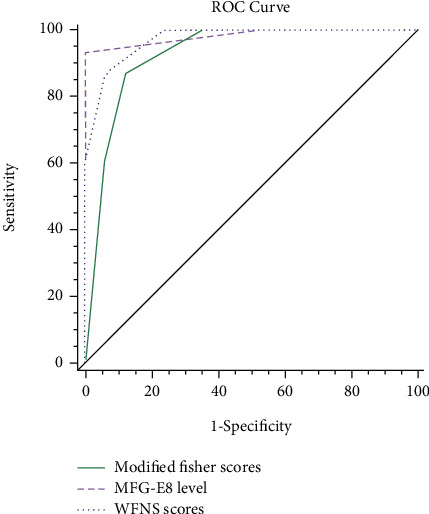
Predictive significance of modified Fisher scores, MFG-E8 level, and WFNS scores in DCI.

The optimal threshold of MFG-E8 was 9314 pg/mL, and its sensitivity, specificity, and Yoden index were 93.3%, 100%, and 0.9333, respectively. The sensitivities of WFNS and modified Fisher scores were 86.67% and 86.67%; the specificities were 94.12% and 88.24; and the Yoden indexes were 0.8078 and 0.7490, respectively (Figure 5). Based on these results, we suggest that the levels of MFG-E8, WFNS scores, and modified Fisher scores can be used in the evaluation of DCI occurrence.

### 3.3. Survival Analysis of MFG-E8 Levels and DCI

**Figure 6:**
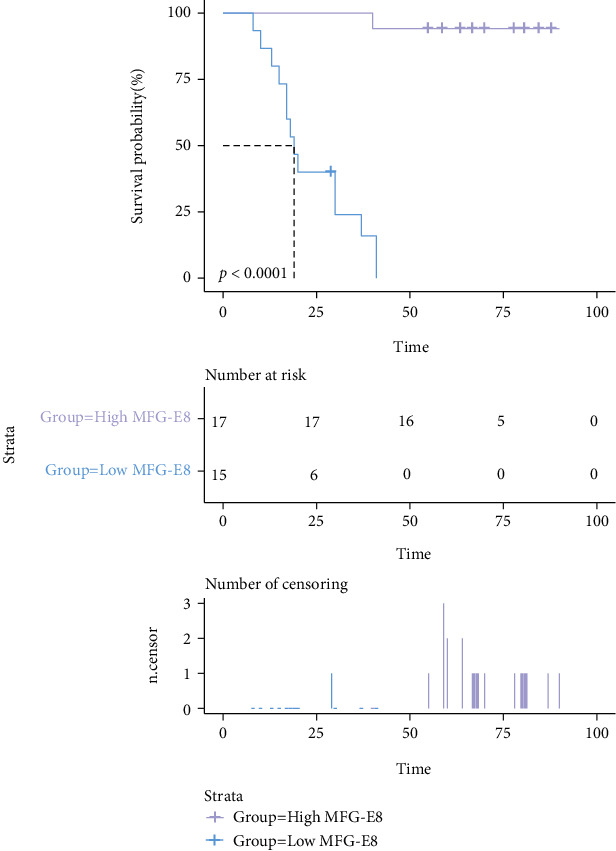
Kaplan–Meier curve of survival rates in patients with DCI.

The Kaplan–Meier curve showed that the 30, 60, and 90-day survival rates (of patients without DCI) in the high-level MFG-E8 group were 100.0% (17/17), 94.1% (10/17), and 94.1% (0/17), respectively. In the low-level group, the survival rates were 24.0% (3/15), 0.0% (0/15), and 0.0% (0/15). The possibility of developing DCI in the high MFG-E8 group was significantly lower than that in the low MFG-E8 group (*p* < 0.001) (Figure 6).

## 4. Discussion

aSAH is one of the most common and critical events of cerebrovascular diseases, representing 5–7% of all strokes [1]. It is caused by the sudden rupture of an intracranial aneurysm, which usually requires surgical treatment. DCI is a common complication in patients with aSAH and appears 3–14 days postoperation [31]. About 30% of patients with aSAH will develop DCI, which contributes significantly to a poor prognosis with treatments being mostly ineffective [6]. The outcomes include permanent neurological damage, disability, and high mortality rates [32, 33]. If the risk of DCI can be predicted in the early stages of aSAH, clinicians can formulate more accurate prognoses, design targeted treatment plans, and optimize the allocation of medical resources. Qualitative or semiquantitative clinical grades such as WFNS and modified Fisher scores are related to the severity of aSAH and are frequently used to predict the occurrence of DCI [21, 23]. However, there are subjective factors in artificial scoring, which may affect the prediction results. Therefore, finding a simpler and more objective biomarker to predict DCI may be more effective in evaluating patient outcomes after aSAH [34, 35]. Unfortunately, few reliable predictors of DCI have been reported [36, 37], and common biomarkers have failed to effectively predict DCI.

It was commonly accepted that DCI was predominantly caused by cerebral vasospasms. In recent years, clinical and animal models have supported the multifactor theory of DCI [12]. Research suggests that inflammation and microthrombosis may play important roles in the pathogenesis of cerebral hemorrhage [10–14]. Lin et al. have shown that sLOX-1 can effectively be used as a biomarker of DCI after aSAH [38]; Ding et al. found that early neuroglobin levels may play a predictive role in the occurrence of DCI [39]. Therefore, biomarkers related to inflammation and microthrombosis could potentially predict the occurrence of DCI in patients with aSAH.

As a multifunctional secretory glycoprotein, MFG-E8 is expressed throughout the body [40]. In the central nervous system, MFG-E8 is mainly expressed by astrocytes and radial glial-like neural stem cells [16, 41], which play important roles in maintaining vascular integrity and reducing inflammation [42, 43]. After an aneurysm rupture, apoptotic vascular endothelial cells synthesize and express MFG-E8, while other types of cells can also increase the level of MFG-E8 by recognizing the binding of integrin *α* V *β* 5 to vascular endothelial cells [44]. In an SAH mouse model, increasing the level of MFG-E8 not only reduced oxidative stress and inflammation but also promoted the phagocytic function of phagocytes and significantly reduced microthrombosis after SAH [45–49]. Therefore, we predicted a plausible relationship between MFG-E8 levels and aSAH.

In a study of specific biomarkers, it was found that related proinflammatory cytokines such as TNF-*α*, IL-1, and IL-6 are involved in the development of DCI [50]. MFG-E8 has been proved to be involved in a variety of biological functions, such as phagocytosis and clearance of apoptotic cells, neovascularization, and reducing inflammation, among others [51, 52]. Gao et al. confirmed that MFG-E8 can effectively attenuate inflammation, reduce brain edema, and improve neurological impairment in an SAH animal model [19, 42]. The neuroprotective effects of MFG-E8 are expected to be achieved through the integrin *β*3/SOCS3/STAT3 pathway. Accordingly, downregulation of MFG-E8 expression by siRNA aggravates the prognosis of SAH [53]. In addition, MFG-E8 reduces inflammation by driving M2 polarization of microglia [42, 54]. Future research should therefore investigate the potential mechanistic targets in the signal pathways that could promote the expression of MFG-E8 for treatment.

In this study, we found that the level of MFG-E8 was highly expressed in patients with aSAH, and the higher the level, the lower the risk of developing DCI. It is suggested that high levels of serum MFG-E8 in patients with aSAH may be related to its anti-inflammatory effects. Additionally, we confirmed that an increase in MFG-E8 level is closely related to an increase in the clinical scores (WFNS and modified Fisher scores) that predict the severity of aSAH, which implies that the severity of bleeding in aSAH patients could be determined by measuring serum MFG-E8. The present study confirmed MFG-E8 as an independent predictor of DCI events, and its predictive ability was similar to that of WFNS and modified Fisher scores. Laboratory examination is more quantitative and objective than clinical evaluation, is more sensitive to small changes in the patient's physical condition, and could aid clinicians in making treatment strategies in a timely manner. According to the Kaplan–Meier survival curves, the incidence of DCI events in the high-level MFG-E8 group was significantly lower than that in the low-level group, and aSAH patients with low levels of MFG-E8 had the highest incidence of DCI within 30 days after operation, implying that these patients require careful monitoring postoperation.

Interestingly, the average ages of both the case and the control groups were more than 55 years, and middle-aged women were more likely to suffer from aSAH than middle-aged men (21 vs. 8, *p* < 0.05). Our comparison of MFG-E8 expression between sexes in the aSAH group yielded different results when analyzed using western blotting and ELISA, which may indicate that ELISA is more sensitive to serum secretory proteins than western blotting. The review on the incidence of SAH by de Rooij et al. is consistent with the results of our study: the incidence of aSAH in women is higher than in men, and the sex difference begins at 55 years of age and increases continuously [55]. We propose that the sex difference may be related to the decrease of pituitary hormone levels, especially estrogen levels in postmenopausal women. The replacement effects of sex hormones have been confirmed in many animal studies [56], though the relationship between the decrease of estrogen level and the expression of MFG-E8 in aSAH requires more investigation.

In summary, MFG-E8 may be used as a potential biomarker to predict the occurrence of DCI after aSAH, and high levels of MFG-E8 may improve the survival rate after DCI. We also unexpectedly found that MFG-E8 levels in patients with unruptured aneurysms were significantly lower than those in patients with ruptured aneurysms. Therefore, we suggest that MFG-E8 may also be used as an indicator of aneurysm rupture, though this will require further investigation. We recognize certain limitations to this study: to our great regret, because of the small sample size, we are unable to carry out multivariate analysis. Besides, the follow-up time is short, and it comes from a single center. Therefore, our study needs to be compared with a multicenter, large sample, and longer follow-up study.

## 5. Conclusions

This study found that there was a correlation between serum MFG-E8 levels and DCI after aSAH. MFG-E8 may be used as a biomarker to predict the occurrence of DCI after aSAH. However, this conclusion needs to be confirmed by multicenter, large sample, and longer follow-up studies.

## Figures and Tables

**Table 1:** Baseline characteristics of the study groups (aSAH and non-aSAH groups).

	aSAH group (*n* = 32)	Non-aSAH group (*n* = 24)	*p* value
Age (years)	56.5 (48.0, 63.0)	60.5 (55.5, 68.0)	0.145
Gender, female	21 (65.6)	8 (33.3)	0.03
MFG-E8 (pg/mL)	11160.9 (6102.0)	3081.0 (2151.1)	<0.001
Aneurysmal diameter (mm)	5.0 (4.0, 6.0)	6.5 (6.0, 8.0)	0.004
Cystic aneurysm	16 (50.0)	15 (62.5)	0.421
Aneurysm in anterior circulation	18 (56.2)	16 (66.7)	0.581
Acute hydrocephalus	17 (53.1)	14 (58.3)	0.789
Intraventricular hemorrhage	21 (65.6)	14 (58.3)	0.591
Alcohol	20 (62.5)	14 (58.3)	0.788
Smoking	13 (40.6)	14 (58.3)	0.28
Hypertension	16 (50.0)	12 (50.0)	1.000
Blood glucose level (mmol/L)	6.1 (1.8)	6.2 (2.5)	0.845
Hs-crp (*μ*g/mL)	19.4 (35.4)	35.7 (60.9)	0.211
LDL (mmol/L)	2.7 (0.8)	2.6 (0.7)	0.742
TG (mmol/L)	1.1 (0.5)	1.2 (0.5)	0.984

**Table 2:** Characteristics of the 32 aSAH patients.

*N*	32
Age (years)	56.5 (48.0, 63.0)
Gender, female	21 (65.6)
MFG-E8 (pg/mL)	11160.9 (6102.0)
Aneurysmal diameter (mm)	5.0 (4.0, 6.0)
Cystic aneurysm	16 (50.0)
Aneurysm in anterior circulation	18 (56.2)
Acute hydrocephalus	17 (53.1)
Intraventricular hemorrhage	21 (65.6)
DCI	15 (46.9)
Alcohol	20 (62.5)
Smoking	13 (40.6)
Hypertension	16 (50.0)
Blood glucose level (mmol/L)	6.1 (1.8)
WFNS scores	
I	7 (21.9)
II	6 (18.8)
III	5 (15.6)
IV	5 (15.6)
V	9 (28.1)
Modified Fisher scores	
I	11 (34.4)
II	6 (18.8)
III	5 (15.6)
IV	10 (31.3)
Hs-crp (*μ*g/mL)	19.4 (35.4)
LDL (mmol/L)	2.7 (0.8)
TG (mmol/L)	1.1 (0.5)

**Table 3:** Comparison of the demographic and clinical data between DCI and non-DCI groups.

	DCI (*n* = 15)	Non-DCI (*n* = 17)	*p* value
Age (years)	52.7 (12.3)	58.7 (9.4)	0.127
Gender, male	3 (20.0)	8 (47.1)	0.147
MFG-E8 (pg/mL)	5882.7 (3162.4)	15818.2 (3771.6)	<0.001
Aneurysmal diameter (mm)	4.7 (1.4)	5.7 (2.0)	0.106
Cystic aneurysm	7 (46.7)	9 (52.9)	1.000
Aneurysm in anterior circulation	8 (53.3)	10 (58.8)	1.000
Acute hydrocephalus	7 (46.7)	10 (58.8)	0.723
Hypertension	4 (26.7)	12 (70.6)	0.032
Intraventricular hemorrhage	10 (66.7)	11 (64.7)	1.000
WFNS scores			<0.001
I	0 (0.0)	7 (41.2)	
II	0 (0.0)	6 (35.3)	
III	2 (13.3)	3 (17.6)	
IV	4 (26.7)	1 (5.9)	
V	9 (60.0)	0 (0.0)	
Modified Fisher scores			<0.001
I	0 (0.0)	11 (64.7)	
II	2 (13.3)	4 (23.5)	
III	4 (26.7)	1 (5.9)	
IV	9 (60.0)	1 (5.9)	

**Table 4:** Univariate logistic regression analysis of DCI in patients with aSAH.

	OR	CI	*p*
Age (years)	0.95	0.88-1.02	0.133
Gender, female	3.56	0.73-17.32	0.116
MFG-E8, <12500 pg/mL	224	12.79-3923.69	<0.001
WFNS 4 to 5	104	8.46-1279.18	<0.001
Modified Fisher scores 3 to 4	48.75	5.99-396.51	<0.001
Hypertension	0.15	0.03-0.71	0.017
Alcohol	1.4	0.33-5.93	0.648
Smoking	1.6	0.39-6.64	0.514
Aneurysm in anterior circulation	0.8	0.2-3.25	0.755
Cystic aneurysm	0.78	0.19-3.13	0.723
Aneurysmal diameter (mm)	0.69	0.44-1.09	0.114
Acute hydrocephalus	0.61	0.15-2.49	0.493
Intraventricular hemorrhage	1.09	0.25-4.71	0.907
Hs-crp (*μ*g/mL)	0.98	0.95-1.01	0.17
Blood glucose level (mmol/L)	0.88	0.59-1.32	0.541
LDL (mmol/L)	1.09	0.44-2.73	0.847
TG (mmol/L)	0.94	0.23-3.84	0.934

## Data Availability

The data that support the findings of this study are available on request from the corresponding author. The data are not publicly available due to patients' privacy or ethical restrictions.

## Abbreviations

MFG-E8: Milk fat globule epidermal growth factor 8
aSAH: Aneurysmal subarachnoid hemorrhage
DCI: Delayed cerebral ischemia
WFNS: World Federation of Neurosurgical Societies
ELISA: Enzyme-linked immunosorbent assay
CT: Computed tomography
CTA: Computed tomography angiography
DSA: Digital subtraction angiography
AUC: Area under the curve
CI: Confidence interval.
